# Supplementary figures and images for: Transcriptomic complexity of the human malaria parasite Plasmodium falciparum revealed by long-read sequencing
Source: PLoS One. 2022 Nov 4;17(11):e0276956. doi: 10.1371/journal.pone.0276956 (PMC9635732; doi:10.1371/journal.pone.0276956)

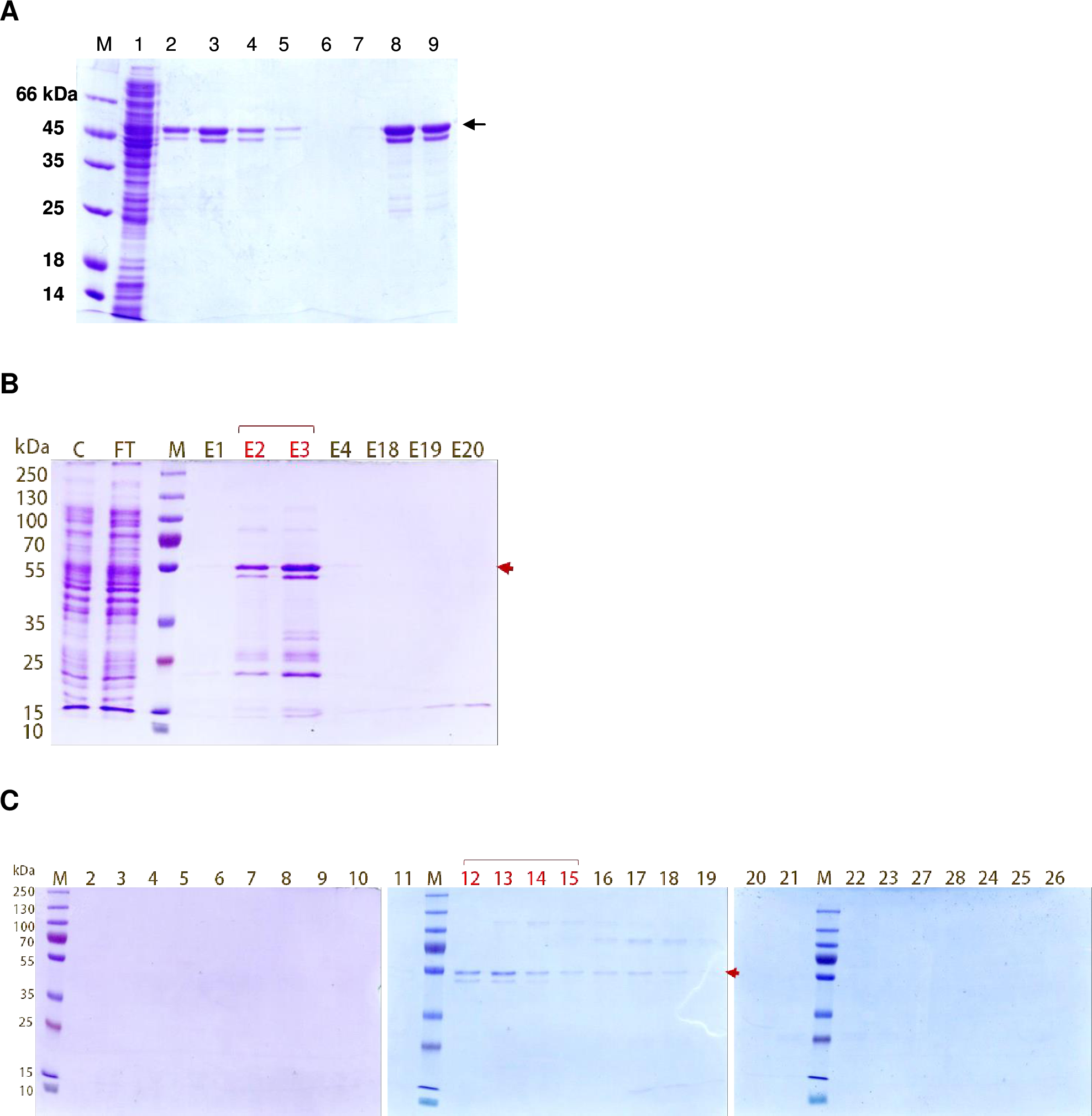

Supplement: S1 Fig — Recombinant protein was expressed in Escherichia coli BL21(DE3) transformed with HseIF4E-eIF4G_x6His plasmid. Protein samples were separated by 12% SDS-PAGE and stained with Coomassie blue. (A) 5′ cap-binding assay by specific elution from aminophenyl-m7GTP (C10-spacer) agarose (Jena Bioscience). The migrations of prestained protein marker bands (Thermo Scientific) are indicated to the left of the lane marked M. Lane 1, crude soluble protein extract; lanes 2–5, m7GTP eluted fractions; lane 6, void volume from NAP-25 Sephadex gel-filtration column; lanes 7–9, fractions 1−3 eluted from gel filtration column. The protein band of the size expected for HseIF4E-eIF4G_x6His fusion protein (46 kDa) is marked by an arrow. (B) HisTrap FF Ni2+-Sepharose purification of HseIF4E-eIF4G_x6His recombinant protein. Lane C, crude soluble extract; lane FT, flow-through of unbound protein; lane M, PageRuler Plus prestained protein marker (Thermo Scientific); lanes E1−E20, imidazole gradient elution fractions. HseIF4E-eIF4G_x6His protein (46 kDa, arrowed) eluted in fractions E2 and E3. (C) Q-Sepharose FF purification. Fractions E2 and E3 from HisTrap FF Ni2+-Sepharose were applied to the column. Lane M, PageRuler Plus prestained protein marker (Thermo Scientific); lanes 2−26, NaCl gradient elution fractions. HseIF4E-eIF4G_x6His protein (46 kDa, arrowed) eluting in fractions 12−15 was used for the enrichment of 5′ capped mRNA. (TIF) [file pone.0276956.s001.tif]

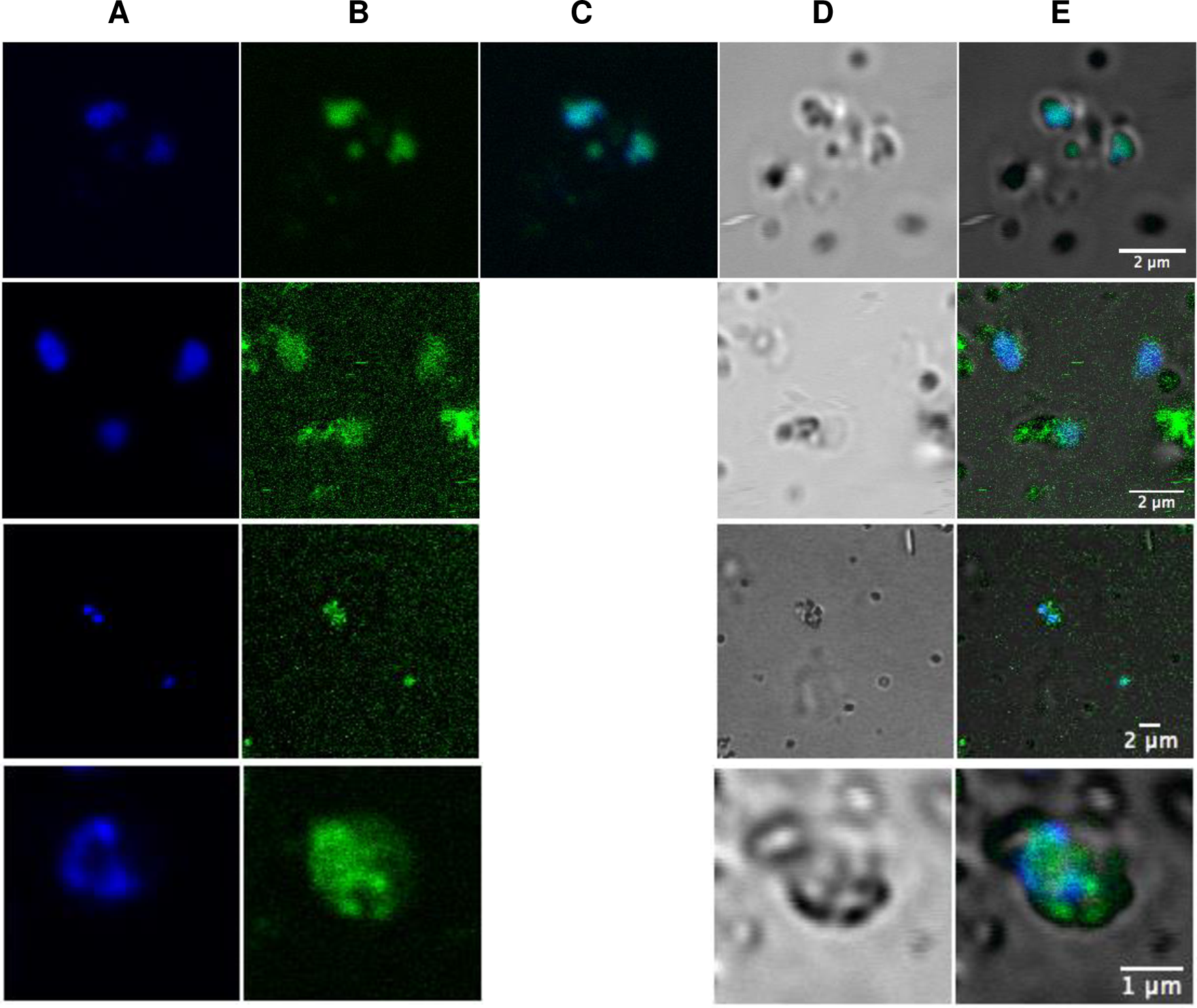

Supplement: S2 Fig — Nuclei were isolated from GAL4-GFP P. falciparum transgenic parasites [80] by differential lysis and centrifugation. Four different fields of view are shown in separate rows, with columns labeled A–E showing different signals of the same field of view. (A) channel 1, Hoechst 33342 signal; (B) channel 2, GFP signal; (C) merged channel 1 and 2; (D) brightfield and (E) merged channel 1,2 and brightfield. Scale bars (2, 2, 2, and 1 μm) are shown in column (E). (TIF) [file pone.0276956.s002.tif]

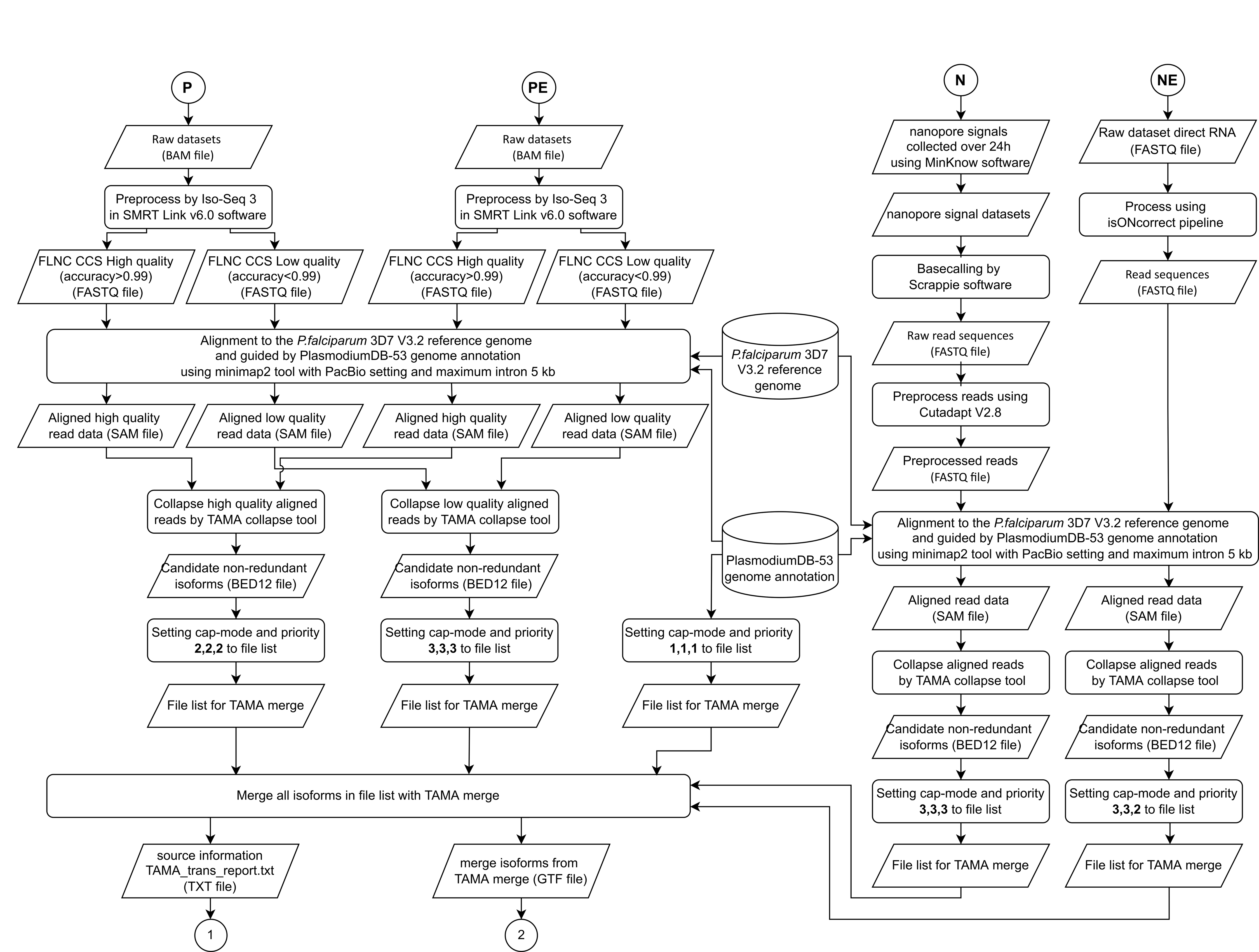

Supplement: S3 Fig — Flowchart shows the data analysis steps used to construct the isoform catalog from raw long-read RNA-Seq data to merged candidate non-redundant isoforms. Raw data sources are shown as P (PacBio data from this study), PE (PacBio data from the Yang et al study [21]), N (Nanopore data from this study), and NE (direct RNA Nanopore data from the Lee et al study [22]). Figure was created using Diagrams.net (available from https://app.diagrams.net/). (TIF) [file pone.0276956.s003.tif]

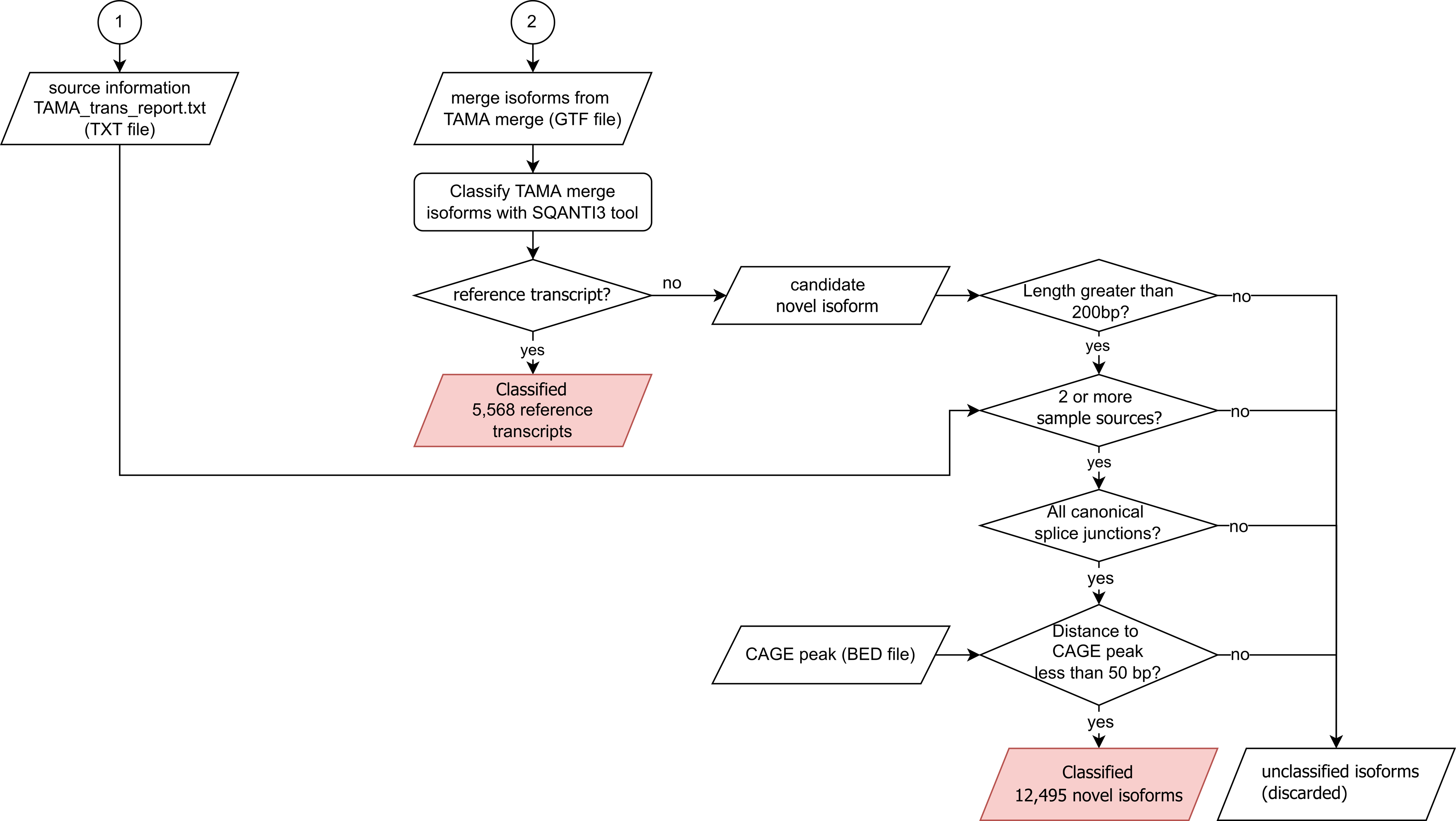

Supplement: S4 Fig — Flowchart shows the final data analysis steps used to construct the isoform catalog. The TAMA merge file outputs of source information (TAMA_trans_report.txt) and merged candidate non-redundant isoforms (TAMA_merge.gtf) were used to construct the final isoform catalog. Figure was created using Diagrams.net (available from https://app.diagrams.net/). (TIF) [file pone.0276956.s004.tif]

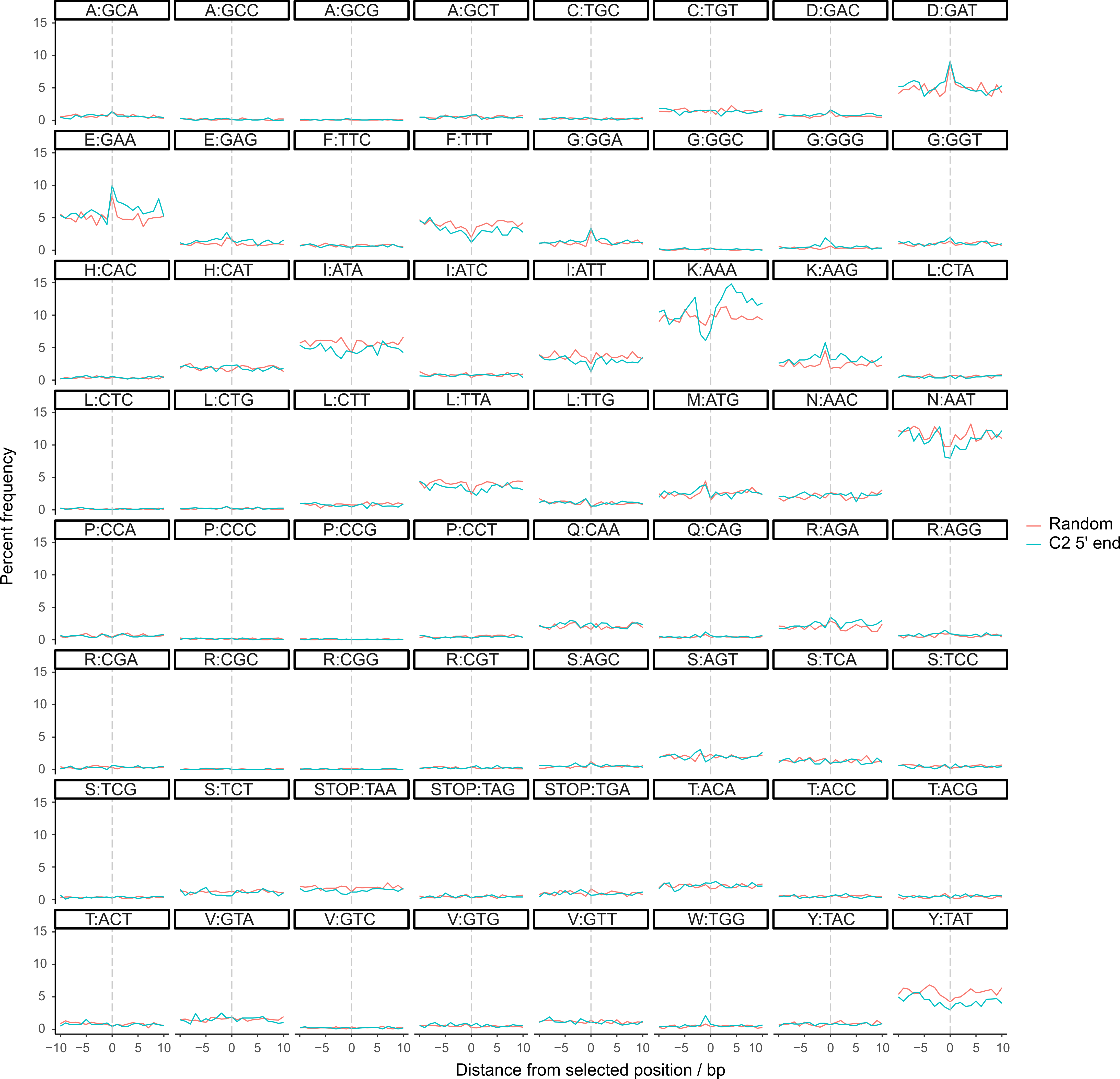

Supplement: S5 Fig — Plots show the percent frequency of each codon in analysis windows (selected Plasmodium falciparum 3D7 v3 genomic positions, 10 bases upstream and downstream) in annotated protein-coding sequence regions. Selected positions corresponding to the 5′ end positions of novel isoforms assigned to the C2 cluster (C2 5′ end) are in blue and randomly selected positions from the same genes are shown in red. (TIF) [file pone.0276956.s005.tif]

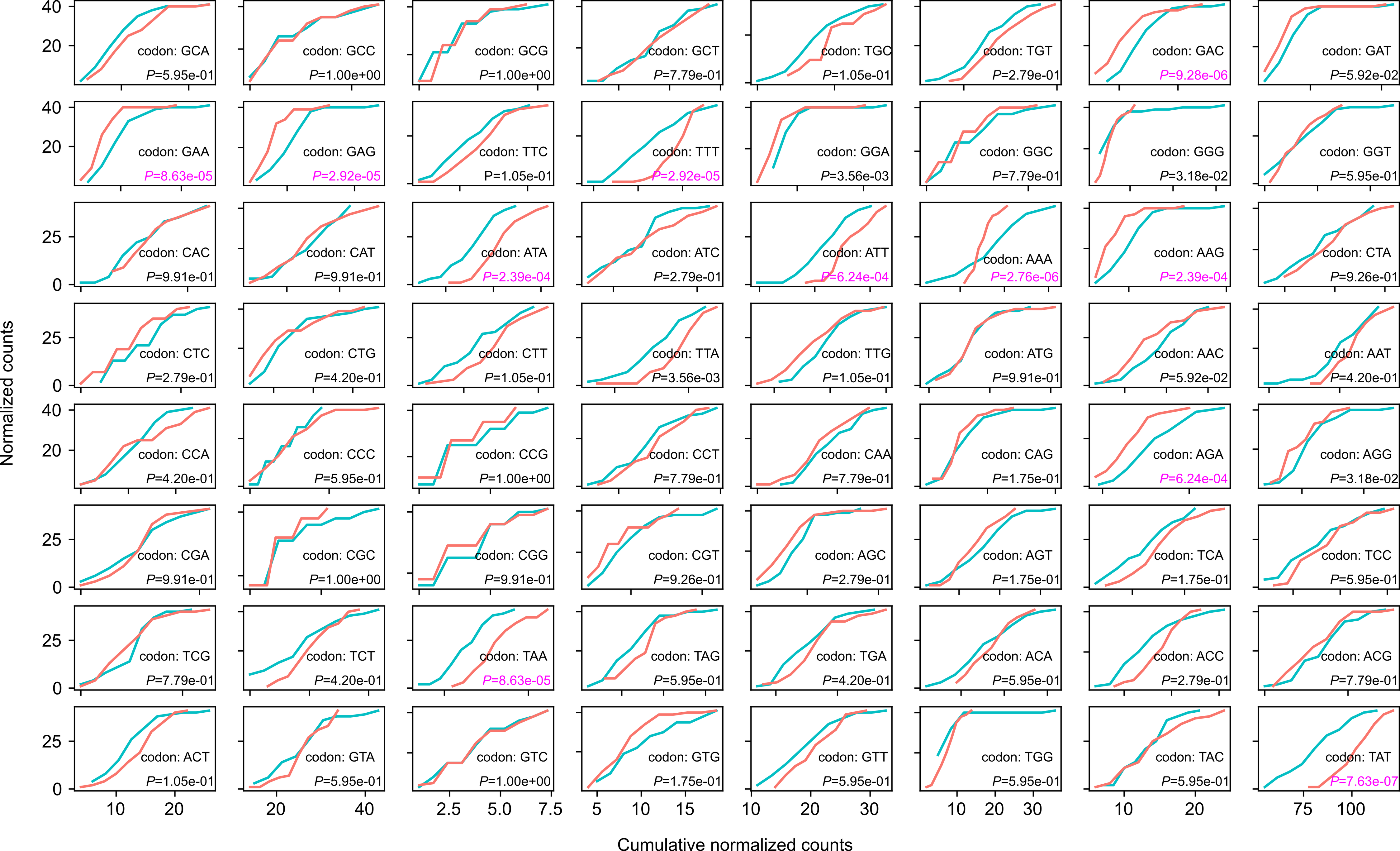

Supplement: S6 Fig — The plots of all 64 codons are shown for analysis windows corresponding to Plasmodium falciparum 3D7 C2 5′ ends (blue) and randomly selected positions (red). The distributions were compared by two-sample, two-sided Kolmogorov-Smirnov test; unadjusted test P-values are shown on each plot and Holm-Bonferroni adjusted P < .05 are indicated in magenta. (TIF) [file pone.0276956.s006.tif]

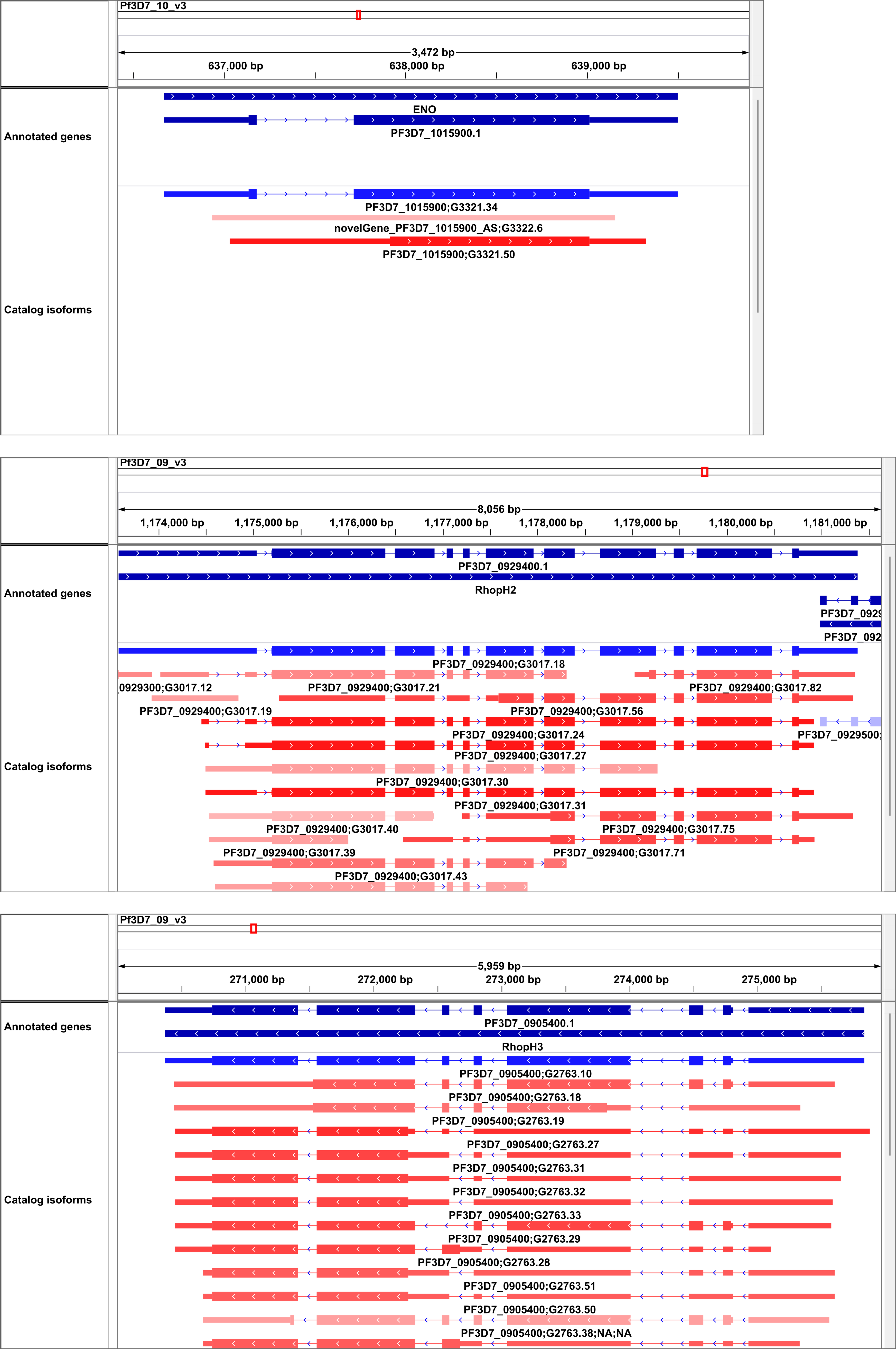

Supplement: S7 Fig — Screenshots from the IGV genome browser [38] are shown for selected regions of Plasmodium falciparum 3D7 chromosome 10 (enolase gene top), and 9 (RhopH2 gene middle and RhopH3 gene bottom). Truncated protein isoforms expressed from these genes were reported previously from two-dimensional differential gel electrophoresis [65]. The structures of PlasmoDB [16] annotated genes and catalog isoforms are shown in the “Annotated genes” and “Catalog isoforms” tracks, respectively (boxes, exons; thick boxes, open reading frames; lines, introns, and arrows, strand orientation). In the "Catalog isoforms" track, reference transcripts are in blue and novel isoforms are in red. The color hue of each isoform reflects the level of support from long-read RNA-seq data (darker, more support). Isoform identifiers (S2 Table) are indicated after the gene identifier. (TIF) [file pone.0276956.s007.tif]
