# Supplementary material for: Transcriptomic complexity of the human malaria parasite Plasmodium falciparum revealed by long-read sequencing
Source: PLoS One. 2022 Nov 4;17(11):e0276956. doi: 10.1371/journal.pone.0276956 (PMC9635732; doi:10.1371/journal.pone.0276956)
Supplement: S1 Text — (DOCX) [file pone.0276956.s018.docx]

>HseIF4E_eIF4G_x6His [gray, peptide linker; HseIF4E, yellow; green, HseIF4G; blue, x6His tag] ttcttgaagacgaaagggcctcgtgatacgcctatttttataggttaatgtcatgataataatggtttcttagacgtcaggtggcacttttcggggaaatgtgcgcggaacccctatttgtttatttttctaaatacattcaaatatgtatccgctcatgagacaataaccctgataaatgcttcaataatattgaaaaaggaagagtatgagtattcaacatttccgtgtcgcccttattcccttttttgcggcattttgccttcctgtttttgctcacccagaaacgctggtgaaagtaaaagatgctgaagatcagttgggtgcacgagtgggttacatcgaactggatctcaacagcggtaagatccttgagagttttcgccccgaagaacgttttccaatgatgagcacttttaaagttctgctatgtggcgcggtattatcccgtgttgacgccgggcaagagcaactcggtcgccgcatacactattctcagaatgacttggttgagtactcaccagtcacagaaaagcatcttacggatggcatgacagtaagagaattatgcagtgctgccataaccatgagtgataacactgcggccaacttacttctgacaacgatcggaggaccgaaggagctaaccgcttttttgcacaacatgggggatcatgtaactcgccttgatcgttgggaaccggagctgaatgaagccataccaaacgacgagcgtgacaccacgatgcctgcagcaatggcaacaacgttgcgcaaactattaactggcgaactacttactctagcttcccggcaacaattaatagactggatggaggcggataaagttgcaggaccacttctgcgctcggcccttccggctggctggtttattgctgataaatctggagccggtgagcgtgggtctcgcggtatcattgcagcactggggccagatggtaagccctcccgtatcgtagttatctacacgacggggagtcaggcaactatggatgaacgaaatagacagatcgctgagataggtgcctcactgattaagcattggtaactgtcagaccaagtttactcatatatactttagattgatttaaaacttcatttttaatttaaaaggatctaggtgaagatcctttttgataatctcatgaccaaaatcccttaacgtgagttttcgttccactgagcgtcagaccccgtagaaaagatcaaaggatcttcttgagatcctttttttctgcgcgtaatctgctgcttgcaaacaaaaaaaccaccgctaccagcggtggtttgtttgccggatcaagagctaccaactctttttccgaaggtaactggcttcagcagagcgcagataccaaatactgtccttctagtgtagccgtagttaggccaccacttcaagaactctgtagcaccgcctacatacctcgctctgctaatcctgttaccagtggctgctgccagtggcgataagtcgtgtcttaccgggttggactcaagacgatagttaccggataaggcgcagcggtcgggctgaacggggggttcgtgcacacagcccagcttggagcgaacgacctacaccgaactgagatacctacagcgtgagctatgagaaagcgccacgcttcccgaagggagaaaggcggacaggtatccggtaagcggcagggtcggaacaggagagcgcacgagggagcttccagggggaaacgcctggtatctttatagtcctgtcgggtttcgccacctctgacttgagcgtcgatttttgtgatgctcgtcaggggggcggagcctatggaaaaacgccagcaacgcggcctttttacggttcctggccttttgctggccttttgctcacatgttctttcctgcgttatcccctgattctgtggataaccgtattaccgcctttgagtgagctgataccgctcgccgcagccgaacgaccgagcgcagcgagtcagtgagcgaggaagcggaagagcgcctgatgcggtattttctccttacgcatctgtgcggtatttcacaccgcatatatggtgcactctcagtacaatctgctctgatgccgcatagttaagccagtatacactccgctatcgctacgtgactgggtcatggctgcgccccgacacccgccaacacccgctgacgcgccctgacgggcttgtctgctcccggcatccgcttacagacaagctgtgaccgtctccgggagctgcatgtgtcagaggttttcaccgtcatcaccgaaacgcgcgaggcagctgcggtaaagctcatcagcgtggtcgtgaagcgattcacagatgtctgcctgttcatccgcgtccagctcgttgagtttctccagaagcgttaatgtctggcttctgataaagcgggccatgttaagggcggttttttcctgtttggtcactgatgcctccgtgtaagggggatttctgttcatgggggtaatgataccgatgaaacgagagaggatgctcacgatacgggttactgatgatgaacatgcccggttactggaacgttgtgagggtaaacaactggcggtatggatgcggcgggaccagagaaaaatcactcagggtcaatgccagcgcttcgttaatacagatgtaggtgttccacagggtagccagcagcatcctgcgatgcagatccggaacataatggtgcagggcgctgacttccgcgtttccagactttacgaaacacggaaaccgaagaccattcatgttgttgctcaggtcgcagacgttttgcagcagcagtcgcttcacgttcgctcgcgtatcggtgattcattctgctaaccagtaaggcaaccccgccagcctagccgggtcctcaacgacaggagcacgatcatgcgcacccgtggccaggacccaacgctgcccgagatctcgatcccgcgaaattaatacgactcactatagggagaccacaacggtttccctctagaaataattttgtttaactttaagaaggagatatacatatggctagcatgactggtggacagcaaatgggtcgggattcaagcttggtaccgagctcggatccactagtaacggccgccagtgtgctggaattcAAAACGGAATCTAATCAGGAGGTTGCTAACCCAGAACACTATATTAAACATCCCCTACAGAACAGATGGGCACTCTGGTTTTTTAAAAATGATAAAAGCAAAACTTGGCAAGCAAACCTGCGGCTGATCTCCAAGTTTGATACTGTTGAAGACTTTTGGGCTCTGTACAACCATATCCAGTTGTCTAGTAATTTAATGCCTGGCTGTGACTACTCACTTTTTAAGGATGGTATTGAGCCTATGTGGGAAGATGAGAAAAACAAACGGGGAGGACGATGGCTAATTACATTGAACAAACAGCAGAGACGAAGTGACCTCGATCGCTTTTGGCTAGAGACACTTCTGTGCCTTATTGGAGAATCTTTTGATGACTACAGTGATGATGTATGTGGCGCTGTTGTTAATGTTAGAGCTAAAGGTGATAAGATAGCAATATGGACTACTGAATGTGAAAACAGAGAAGCTGTTACACATATAGGGAGGGTATACAAGGAAAGGTTAGGACTTCCTCCAAAGATAGTGATTGGTTATCAGTCCCACGCAGACACAGCTACTAAGAGCGGCTCCACCACTAAAAATAGGTTTGTTGTTGGTGGAGGAGGTTCTGGAGGCGGAGGTACCGGTGGCGGAGGTAGCAGGAGACGGAAAATTAAGGAGCTAAATAAGAAGGAGGCTGTTGGAGACCTTCTGGATGCCTTCAAGGAGGCGAACCCGGCAGTACCAGAGGTGGAAAATCAGCCTCCTGCAGGCAGCAATCCAGGCCCAGAGTCTGAGGGCAGTGGTGTGCCCCCACGTCCTGAGGAAGCAGATGAGACCTGGGACTCAAAGGAAGACAAAATTCACAATGCTGAGAACATCCAGCCCGGGGAACAGAAGTATGAATATAAGTCAGATCAGTGGAAGCCTCTAAACCTAGAGGAGAAAAAACGTTACGACCGTGAGTTCCTGCTTGGTTTTCAGTTCATCTTTGCCAGTATGCAGAAGCCAGAGGGATTGCCACATATCAGTGACGTGGTGCTGGACAAGGCCAATAAAACACCACTGCGGCCACTGGATCCCACTAGACTACAAGGCATAAATTGTGGCCCAGACTTCACTCCATCCTTTGCCAACCTTGGCCATCACCATCATCACCACTGActcgagcagatccggctgctaacaaagcccgaaaggaagctgagttggctgctgccaccgctgagcaataactagcataaccccttggggcctctaaacgggtcttgaggggttttttgctgaaaggaggaactatatccggataa
